# Nuts and Cardiovascular Disease Outcomes: A Review of the Evidence and Future Directions

**DOI:** 10.3390/nu15040911

**Published:** 2023-02-11

**Authors:** Andrea J. Glenn, Dagfinn Aune, Heinz Freisling, Noushin Mohammadifard, Cyril W. C. Kendall, Jordi Salas-Salvadó, David J. A. Jenkins, Frank B. Hu, John L. Sievenpiper

**Affiliations:** 1 Department of Nutrition, Harvard T.H. Chan School of Public Health, Boston, MA 02115, USA; 2 Department of Nutritional Sciences, Temerty Faculty of Medicine, University of Toronto, Toronto, ON M5S 1A8, Canada; 3 Toronto 3D Knowledge Synthesis and Clinical Trials Unit, Clinical Nutrition and Risk Factor Modification Centre, St. Michael’s Hospital, Toronto, ON M5C 2T2, Canada; 4 Department of Epidemiology and Biostatistics, School of Public Health, Imperial College London, London SW7 2AZ, UK; 5 Department of Nutrition, Oslo New University College, 0372 Oslo, Norway; 6 Department of Endocrinology, Morbid Obesity and Preventive Medicine, Oslo University Hospital, 0586 Oslo, Norway; 7 Nutrition and Metabolism Branch, International Agency for Research on Cancer (IARC-WHO), 69366 Lyon, France; 8 Isfahan Cardiovascular Research Center, Cardiovascular Research Institute, Isfahan University of Medical Sciences, Isfahan, Iran; 9 College of Pharmacy and Nutrition, University of Saskatchewan, Saskatoon, SK S7N 5E5, Canada; 10 Department of Biochemistry & Biotechnology, School of Medicine, Institut d’Investigacions Sanitàries Pere i Virgili, Rovira i Virgili University, 43204 Reus, Spain; 11 CIBER Fisiopatología de la Obesidad y Nutrición (CIBEROBN), Instituto de Salud Carlos III, 28029 Madrid, Spain; 12 Li Ka Shing Knowledge Institute, St. Michael’s Hospital, Toronto, ON M5B 1T8, Canada; 13 Division of Endocrinology and Metabolism, Department of Medicine, St. Michael’s Hospital, Toronto, ON M5C 2T2, Canada; 14 Department of Medicine, Temerty Faculty of Medicine, University of Toronto, Toronto, ON M5S 1A8, Canada; 15 Channing Division of Network Medicine, Department of Medicine, Brigham and Women’s Hospital and Harvard Medical School, Boston, MA 02115, USA; 16 Department of Epidemiology, Harvard T.H. Chan School of Public Health, Boston, MA 02115, USA

**Keywords:** tree nuts, peanuts, nutrition, cardiovascular diseases, review

## Abstract

Nuts are nutrient-rich foods that contain many bioactive compounds that are beneficial for cardiovascular health. Higher consumption of nuts has been associated with a reduced risk of several cardiovascular diseases (CVD) in prospective cohort studies, including a 19% and 25% lower risk of CVD incidence and mortality, respectively, and a 24% and 27% lower risk of coronary heart disease incidence and mortality, respectively. An 18% lower risk of stroke mortality, a 15% lower risk of atrial fibrillation, and a 19% lower risk of total mortality have also been observed. The role of nuts in stroke incidence, stroke subtypes, peripheral arterial disease and heart failure has been less consistent. This narrative review summarizes recommendations for nuts by clinical practice guidelines and governmental organizations, epidemiological evidence for nuts and CVD outcomes, nut-containing dietary patterns, potential mechanisms of nuts and CVD risk reduction, and future research directions, such as the use of biomarkers to help better assess nut intake. Although there are still some uncertainties around nuts and CVD prevention which require further research, as summarized in this review, there is a substantial amount of evidence that supports that consuming nuts will have a positive impact on primary and secondary prevention of CVD.

## 1. Introduction

Cardiovascular diseases (CVD) are the leading cause of death worldwide and a major cause of premature mortality, causing an estimated 31% of all deaths globally [1]. Major modifiable risk factors for CVD include smoking, harmful alcohol use, physical inactivity, unhealthy diets, abdominal obesity, hypertension, dyslipidemia, high fasting glucose and diabetes, and kidney dysfunction [2]. Furthermore, there are many downstream complications of CVD that can significantly impact quality of life and cause disability and death, including dementia, peripheral arterial disease (PAD), heart failure (HF), kidney disease, frailty and poor aging, among others [3].

Despite a decline in CVD in several regions around the globe, it remains a major threat to public health as the absolute numbers continue to increase, with prevalent cases of total CVD nearly doubling from 1990 to 2019 [4]. These numbers are expected to increase even further in upcoming years due to population growth and aging. For example, a recent analysis in the United States projects large future increases in CVD risk factors and CVD prevalence (e.g., 31% increase in ischemic heart disease and 34% increase in stroke) by 2060 [5]. According to the Global Burden of Diseases (GBD) Study, unhealthy diets are the greatest contributor to premature morbidity and mortality worldwide, including CVD mortality [6]. The main dietary risk factors attributable to the global burden of diseases include diets low in whole grains, fruit, nuts/seeds, and vegetables and diets high in sodium and processed meat [7]. Tree nuts and peanuts, one of the top dietary risk factors noted by the GBD study, may be particularly beneficial for CVD prevention due to their bioactive components. Of note, peanuts are botanically defined as legumes; however, they have a similar nutrient composition and culinary use as tree nuts and are, therefore, usually included as nuts when estimating total nut intake [8]. The bioactive components of tree nuts and peanuts include their macronutrient, fat-soluble bioactive, fiber, vitamin, mineral and phenolic content [9]. Specifically, fat-soluble bioactives such as their fatty acid content (monounsaturated and polyunsaturated fatty acids), fiber, magnesium, tocopherols and tocotrienols, phytosterols, sphingolipids, carotenoids, chlorophylls and alkyl phenols, and phenolic compounds (including flavonoids, phenolic acids, stilbenes, lignans, among others) all likely contribute to their cardiovascular health-promoting effects [9].

In this narrative review, we describe the importance given to nuts in clinical practice guidelines, the evidence we have in relation to the beneficial effects of frequent consumption of nuts in the prevention of CVD, as well as the possible mechanisms involved. However, we also emphasize the gaps that exist in the literature and discuss the possible studies that we should develop in the future to increase the level of evidence and establish recommendations.

## 2. Nuts in Clinical Practice Guidelines for Cardiovascular Risk Reduction

Given their cardiovascular health-promoting properties, tree nuts and peanuts are recognized by several international health organizations for cardiovascular risk reduction for both primary and secondary prevention. Table 1 highlights the recommendations from cardiovascular clinical practice guidelines (CPGs), including the Canadian Cardiovascular Society, Joint British Societies for the Prevention of Cardiovascular Disease, the Australian Heart Foundation, the American Heart Association and the European Society of Cardiology [10,11,12,13,14]. Nuts are recommended as a healthy plant protein and fat source that should be frequently consumed to lower low-density lipoprotein cholesterol (LDL-C), improve the overall lipoprotein profile and decrease CVD risk.

## 3. Regulated Nut Health Claims Allowed for Cardiovascular Risk Reduction

To encourage the consumption of foods that may be beneficial for health, governmental agencies review the evidence and approve health claims that can provide consumers with reliable information about the relationship between the consumption of food and a specific health benefit. Several regulated disease risk reduction health claims have been approved for nuts and CVD by the U.S. Food and Drug Administration (FDA), the European Food Safety Authority (EFSA) and Food Standards Australia New Zealand (FSANZ), as shown in Table 2 [15,16]. Health Canada has not approved a disease risk reduction claim for nuts. The FDA and FSANZ both approved a health claim regarding nuts and CVD risk reduction, particularly for the LDL-C lowering effects of nuts. However, EFSA did not approve this type of claim and noted that, specifically for walnuts, the evidence provided did not establish that the consumption of walnuts had an effect on LDL-C beyond what could be expected from their fatty acid composition [17]. EFSA, however, established that a cause-and-effect relationship between the consumption of walnuts and the improvement of endothelium-dependent vasodilation exists and has approved a health claim for this finding (Table 2) [17]. 

## 4. Nuts and Cardiovascular Disease Outcomes

**Figure 1:**
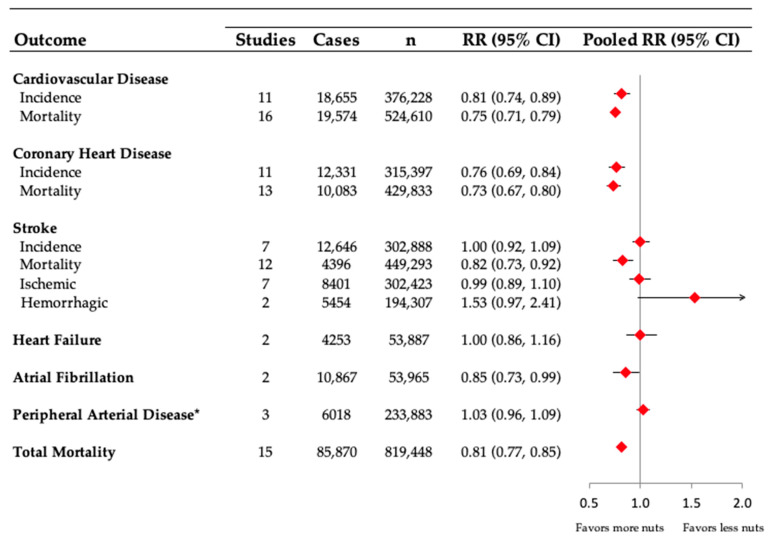
Summary of the pooled effect estimates of prospective cohort studies assessing the association between high and low consumption of nuts and risk of cardiovascular disease outcomes and total mortality. Figure adapted from Figure 2 in [21]. Pooled risk estimate is represented by the diamond. * To obtain summary estimates for peripheral arterial disease, we used generic inverse variance (fixed effects) to pool the natural log-transformed RRs of the extreme quantiles. Abbreviations: CI = confidence intervals; RR = relative risk.

The scientific study of the role of nuts in preventing CVD started over 30 years ago, first with a discovery that individuals in the Adventist Health Study who consumed nuts more frequently (more than four times per week) had fewer coronary heart disease (CHD) events compared to those who consumed nuts less than once per week [18]. Investigators believed that this CHD risk reduction might be related to the favorable fatty acid profile of nuts. Researchers from the same group then assessed if consuming walnuts (20% of calories through replacing other fatty foods, meat and oils, margarine and butter) would affect blood lipids in healthy individuals [19]. They found that incorporating a moderate amount of walnuts in the diet decreased levels of total cholesterol (TC) and LDL-C [19]. The favorable modification of the lipid profile by frequent nut consumption has been confirmed in additional trials and systematic reviews and meta-analyses [20]. The role of nuts in preventing CVD outcomes in prospective cohort studies has also been extensively studied. Below we describe this evidence related to total CVD, CHD, stroke, HF, atrial fibrillation (AF), PAD and total mortality. The definition of nuts includes tree nuts, peanuts and seeds (sunflower, pumpkin, etc.) as a culinary definition for this review. An overview of the pooled summary data for each outcome is included in Figure 1.

### 4.1. Total Cardiovascular Disease

Total CVD is a composite outcome of CVD incidence (including only nonfatal or a combination of nonfatal and fatal outcomes of different CVD outcomes) or may include CVD mortality outcomes only, which is a composite of different fatal CVD endpoints. A recent systematic review and meta-analysis of prospective cohort studies that were commissioned to update the clinical practice guidelines for nutrition therapy for the European Association for the Study of Diabetes (EASD) found that in three cohort comparisons (including 210,839 participants and 14,136 events), high consumption of nuts was associated with a 15% lower risk of CVD incidence (relative risk [RR] = 0.85, 95% confidence intervals [CI]: 0.80–0.91) compared to low consumption [22]. In 15 cohort comparisons (including 413,797 participants and 14,475 events), high consumption of nuts was associated with a 23% lower risk of CVD mortality (RR = 0.77, CI: 0.72–0.82) compared to low consumption [22]. The certainty of the evidence was assessed using the Grading Recommendations Assessment, Development and Evaluation (GRADE). The GRADE for CVD incidence was low quality due to a downgrade for indirectness (i.e., how applicable the evidence is to the general population) but an upgrade for a dose-response gradient. The GRADE for CVD mortality was moderate quality due to an upgrade for a dose-response gradient. The dose-response analysis showed that the reduction in risk of CVD incidence was observed up to 10 g/day with no further reduction in risk for higher consumption. For CVD mortality, there was a greater reduction in risk at 15–20 g/day, with no further reduction with higher consumption. When assessing different types of nuts, tree nuts, peanuts and walnuts were all associated with a 13–19% lower risk of CVD incidence, whereas the association for peanut butter was not significant (RR = 0.98, CI: 0.93–1.03). For CVD mortality, only peanuts have been analyzed, and higher consumption was associated with a lower risk of CVD mortality compared to low consumption [22]. The lack of association with peanut butter may be in part because many peanut butters on the market have added salt, fully hydrogenated oils or oils such as palm oil that can increase the saturated fat content, which could negatively impact their health benefits as compared with whole sources of peanuts; further research on natural peanut butter may provide additional insight into this hypothesis. 

A more recent 2022 umbrella review assessing the role of nuts in preventing several chronic diseases found similar associations with CVD incidence and mortality [21]. For CVD incidence, 11 cohort comparisons were assessed (including 376,228 participants and 18,655 events), and a 19% lower risk was observed comparing high to low consumption (RR = 0.81, CI: 0.74–0.89) and 21% lower risk when assessing associations per serving (28 g/day) (RR = 0.79, CI: 0.70–0.89). For CVD mortality, 16 cohort comparisons were included (524,610 participants and 19,574 cases), and a 25% lower risk comparing high to low consumption (RR = 0.75, CI: 0.71, 0.79) was observed and a 6% lower risk when assessing nut intake by 28 g/day (RR = 0.94, CI: 0.93, 0.96) [21]. This umbrella review also assessed dose-response relationships with CVD mortality and found that the optimal intake levels of nuts are ~15–20 g/day and that there were limited further benefits of consuming up to one serving (28 g/day), similar to the earlier meta-analysis [22]. However, it should be mentioned that the high end of nut consumption across most cohorts is typically one serving (28 g)/day, and little is known about the dose-response relationship with higher intakes.

### 4.2. Coronary Heart Disease

Similar to total CVD, CHD incidence may include fatal and nonfatal events, whereas CHD mortality includes CHD mortality outcomes only. The systematic review and meta-analysis conducted to update the EASD nutrition therapy guidelines assessed 7 cohort comparisons (including 275,812 participants and 12,654 cases) and found that high nut consumption was associated with an 18% lower risk of CHD incidence (RR = 0.82, CI: 0.69–0.96) compared to low consumption [22]. For CHD mortality, 13 cohort comparisons were included (396,014 participants and 7877 cases), and high consumption of nuts was associated with a 24% lower risk (RR = 0.76, CI: 0.67–0.86) compared to low consumption. The certainty of evidence using GRADE was very low for CHD incidence owing to downgrades for inconsistency (i.e., unexplained heterogeneity), indirectness and imprecision (i.e., the minimally important difference for the clinical benefit [considered RR = 0.95 in this meta-analysis]), and an upgrade for a dose-response gradient. The certainty of the evidence was moderate for CHD mortality, owing to an upgrade for a dose-response gradient. When assessing different types of nuts, tree nuts, peanuts and walnuts were all associated with a 15–23% lower risk of CHD incidence, and similar to CVD incidence, no association was reported for peanut butter consumption (RR = 1.00, CI: 0.94–1.07). The findings were also similar between CVD and CHD mortality, where peanut consumption was inversely associated with the risk of CHD mortality. Comparable to CVD mortality, the CHD mortality dose-response analysis showed greater reductions in risk at around 15–20 g/day [13,22]. The 2022 umbrella review showed that for CHD incidence (including 12 cohort comparisons with 315,397 participants and 12,331 events), there was a 24% lower risk comparing high to low consumption (RR = 0.76, CI: 0.69–0.84) and 25% lower risk when assessing per servings associations of 28 g/day (RR = 0.75, CI: 0.64–0.88) [21]. For CHD mortality, 13 cohort comparisons were included (429,833 participants and 10,083 cases), and a 27% lower risk comparing low to high consumption (RR = 0.73, CI: 0.67, 0.80) was observed and a 6% lower risk when assessing nut intake by 28 g/day (RR = 0.94, CI: 0.93, 0.96) [21].

### 4.3. Stroke

Stroke outcomes include stroke incidence and mortality, and the main stroke subtypes, ischemic and hemorrhagic stroke. The association between nut consumption and stroke risk has been less consistent than that observed for total CVD and CHD. For stroke incidence, the EASD systematic review and meta-analysis included 7 cohort comparisons (of 302,888 participants and 12,646 events) and found no associations when comparing high to low consumption (RR = 1.00, CI: 0.92–1.09) [22]. In contrast, for stroke mortality, 12 cohort comparisons were analyzed (including 351,618 participants and 2332 cases) and comparing high vs. low categories of nut consumption was associated with a 17% lower risk (RR = 0.83, CI: 0.75–0.93). The certainty of evidence using GRADE was very low for stroke incidence owing to downgrades for indirectness and imprecision, and low for stroke mortality, owing to downgrades for imprecision but an upgrade for a dose-response gradient. Regarding specific types of nuts, peanut consumption was associated with a lower risk of stroke incidence and mortality, but other nut types and peanut butter were not significantly associated with either outcome. For stroke subtypes, no associations were seen with ischemic stroke (RR = 0.99, CI: 0.89–1.10 in 7 cohort comparisons including 302,423 participants and 8401 cases) or hemorrhagic stroke (RR = 1.02, CI: 0.77–1.34 in 5 cohort comparisons including 188,750 participants and 3088 cases) [22]. 

Another meta-analysis including 11 cohort studies (9272 stroke cases) and 396,768 participants also reported an inverse association in the high vs. low analysis (RR = 0.89, CI: 0.82–0.97), but not in the linear dose-response analysis (RR = 0.93, CI: 0.83–1.05); however, there was some indication of a non-linear J-shaped association with a reduction in risk up to approximately 10–15 g/day, but a slight positive association at 30 g/day [23]. There was no indication of an increased risk at high intakes when stroke incidence and stroke mortality were analyzed separately, suggesting that the direct association at high nut doses observation could be an artefact. When subtypes of nuts were examined, no association was observed for tree nuts in relation to the risk of stroke in the high vs. low and dose-response analyses, while slight inverse associations were observed for peanuts [23], which were both similar to those reported in the EASD meta-analysis [22]. It is unclear whether these differences in results between subtypes are real or simply because of chance variation due to the few studies available. Given largely overlapping confidence intervals between summary estimates, it is possible that chance variation is playing a role; therefore, further studies are needed. The 2022 umbrella review findings were also similar: no association was seen with stroke incidence (RR = 1.00, CI: 0.92–1.09) in 7 cohort comparisons, including 302,888 participants and 12,646 cases, with an inverse association seen with stroke mortality (RR = 0.82, CI: 0.73–0.92) in 12 cohort comparisons including 449,293 participants and 4398 events [21]. Although results regarding nut consumption and stroke risk have been more variable than for CHD, it seems there may be a modest inverse association between higher nut intake and stroke risk. Most of the individual studies may not have been sufficiently powered to detect an association. Nonetheless, a possible reason for the weaker association between nut consumption and stroke than for CHD could be the fact that many nuts are salted. Dietary salt consumption is one of the main determinants for elevated blood pressure, and it is possible that adding salt to nuts could dilute some of the benefits they have, particularly for stroke, similar to the possible reasons for no association seen with peanut butter and several CVD outcomes. 

### 4.4. Heart Failure

Few prospective cohort studies have assessed the role of nut consumption in preventing one of the major complications of CVD, HF. The EASD systematic review and meta-analysis included two cohort studies with 53,877 participants and 4253 cases, and for high vs. low categories of nut consumption, the RR was 1.00 (CI: 0.86–1.16) [22]. The certainty of evidence by GRADE was very low for HF, owing to downgrades due to the risk of bias (i.e., study quality as assessed by the Newcastle Ottawa Scale), indirectness and imprecision. Similarly, the 2022 umbrella review included the same cohorts, and the same effect estimates were observed [21]. Interestingly, a large prospective Swedish Cohort study of 61,364 adults observed a non-linear inverse association with the risk of HF and a 12% reduction in risk (HR = 0.88, CI: 0.79–0.99) with consumption of nuts 1–2 times per week with 17 years of follow-up [24]. 

### 4.5. Atrial Fibrillation

Similar to HF, fewer prospective cohort studies have examined the role of nuts in preventing this important risk factor for stroke and HF. The EASD systematic review and meta-analysis assessed two prospective cohort studies, including 53,965 participants and 10,867 cases [22]. Comparing high vs. low consumption of nuts, there was a 15% lower risk of AF (RR = 0.85, CI: 0.73–0.99), similar to the 2022 umbrella review [21]. The certainty of the evidence was very low, owing to downgrades due to indirectness and imprecision [22]. Similar to HF, the Swedish Cohort study also found a non-linear inverse relationship with the risk of AF and consumption of nuts 3 or more times per week was associated with an 18% reduced risk of AF [24]. 

### 4.6. Peripheral Arterial Disease

Few studies have assessed the association between nut consumption and the risk of PAD [25]. In the Atherosclerosis Risk in Communities Study, 14,082 men and women were followed for 20 years, and 1569 incident cases of PAD were identified. There was no clear association between the frequency of nut consumption and the risk of PAD, and the HR was 1.04 (CI: 0.89–1.23) when comparing an intake of ≥2/week vs. almost never [26]. In an analysis from the Women’s Health Initiative, including 138,506 postmenopausal women and 1036 PAD cases identified during 19 years of follow-up, there was no association between higher consumption of nuts and seeds and PAD (highest vs. lowest quartile of nuts and seeds consumption was 0.93 [CI: 0.78–1.10]) [27]. Similarly, in a combined analysis of 38,823 women in the Swedish Mammography Cohort and 45,472 men in the Cohort of Swedish Men (aged 45–83 years) with 22 years follow-up and 3413 PAD cases, there was no clear association between intake of nuts and PAD risk, and the HR was 1.05 (CI: 0.89–1.24) for the highest vs. lowest category of intake [28]. We pooled the data from the 3 cohorts, and the RR was 1.03 (CI: 0.96–1.09), also highlighting no clear association between nut consumption and the risk of PAD (Figure 1). However, we cannot exclude the possibility that there may be a U-shaped association between nut consumption and PAD. 

### 4.7. Total Mortality

The 2022 umbrella review also assessed the association between nut consumption and overall total mortality. In 16 cohort comparisons (including 819,448 participants and 85,870 deaths), both high vs. low categories of nut consumption (RR = 0.81, CI: 0.77, 0.85) and per serving (28 g/day, RR = 0.78, CI: 0.72, 0.84) were associated with lower risk of total mortality [21]. Similar to total CVD, the dose-response analyses indicated optimal intake levels were approximately ~15–20 g/day, with limited further benefits up to 28 g/day. In addition, a meta-analysis of 15 cohort studies indicated that 4.4 million premature deaths in the Americas, Europe, Southeast Asia and Western Pacific would be attributable to a nut intake below 20 g/day [23]. 

### 4.8. Change in Nut Intake and Substitution Analyses

Changes in nut intake over time and the substitution of nuts for other dietary factors have also been associated with a lower risk of CVD. For instance, Liu et al. examined the association between 4-year changes in nut consumption and risk of CVD outcomes in the subsequent 4 years in the Health Professionals Follow-up Study (HPFS) and Nurses’ Health Study (NHS) I and II [29]. They found that a per ½ serving/day increase in total nut consumption was associated with a lower risk of CVD (HR = 0.92, CI: 0.86–0.98), CHD (HR = 0.94, CI: 0.89–0.99) and stroke (HR = 0.89, CI: 0.83–0.95) and those that decreased their nut consumption over time had an increased risk of CVD, CHD and stroke. The ½ serving/day increase in consumption for a different type of nuts showed that most nut types were associated with a lower risk of CVD outcomes, with walnuts showing the strongest association and peanut butter showing no association. The researchers also examined the substitution effect per ½ serving/day of nuts and found that replacing red meat, processed meat, refined grains, French fries and dessert with nuts was associated with a lower risk of CVD, CHD and stroke [29]. In another analysis of HPFS and NHS, substituting both unprocessed and processed red meat for nuts was associated with the greatest reduction in total mortality [30]. 

## 5. Healthy Dietary Patterns That Contain Nuts

Several healthy dietary patterns that are recommended in CVD clinical practice guidelines contain nuts as a key food component. These include dietary patterns such as the Mediterranean, Nordic, Dietary Approaches to Stop Hypertension (DASH), vegetarian and Portfolio diets. Each of these dietary patterns has been shown to lower important CVD risk factors in RCTs and is associated with a lower risk of CVD in prospective cohort studies [31,32,33,34,35,36,37,38]. A healthy Mediterranean diet including nuts was also assessed with CVD endpoints in the landmark Prevención con Dieta Mediterránea (PREDIMED) trial. In this trial, over 7000 high-risk individuals for CVD were randomly assigned to an energy-unrestricted Mediterranean diet supplemented with either extra virgin olive oil (EVOO) or mixed nuts or the control diet (advice to curtail all types of fat) [39]. After approximately 5 years, both the Mediterranean diet groups supplemented with EVOO and nuts had a 30% reduction in CV events, mainly through reductions in stroke. Further analyses of the PREDIMED study also found a reduced risk of PAD both in the EVOO (HR = 0.36, CI: 0.21–0.65) and in the nuts group (HR = 0.54, CI: 0.32–0.92) when compared to the control group [40]. Due to the design of the PREDIMED study, it is not possible to separate the impact of nuts (or EVOO) from that of other dietary recommendations given to increase adherence to a Mediterranean diet, as well as with other healthy dietary patterns, and it is possible that other components of the diet could contribute in part to these findings. 

## 6. Mechanisms Related to Nuts and Cardiovascular Risk Reduction

There are several mechanisms by which nuts can lower the risk of developing CVD, such as through positively impacting intermediate cardiovascular risk factors, including blood lipids, blood pressure, inflammation, and markers of glycemic control, among others. Nuts are rich in unsaturated fatty acids, plant protein, phytosterols, fiber, some minerals (including potassium, calcium and magnesium), vitamins (vitamin E and B6) and phenolic and bioactive compounds, all of which may contribute to their CV health-promoting benefits [9]. A systematic review and meta-analysis of 61 trials of tree nut consumption found that at a median dose of 56 g/day, TC, LDL-C, apolipoprotein B (ApoB) and triglycerides were significantly lowered, with no effect on high-density lipoprotein-cholesterol (HDL-C). A dose-dependent effect was also reported, with stronger effects seen for TC and LDL-C with nut intake over 60 g/day [20]. Importantly, the type of nut did not appear to be important for the cholesterol-lowering results observed [20]. The effects on blood pressure and inflammation have been less consistent than those observed for blood lipids, with the same systematic review and meta-analysis finding no significant effects of tree nut consumption on blood pressure and C-reactive protein [20]. Other meta-analyses have conversely shown that nut consumption, particularly pistachios, does have a modest blood pressure lowering effects in people without type 2 diabetes [41], with another meta-analysis finding that almond consumption lowered diastolic blood pressure [42]. This finding of stronger effects on blood lipids than on blood pressure is consistent with the studies showing that LDL-C and ApoB are causal in the development of CHD [43], while elevated blood pressure is a greater risk factor for stroke [44] and may explain the more consistent finding with a lower risk of CHD than with stroke seen in the prospective cohort studies described earlier. Furthermore, a systematic review and meta-analysis that included 12 trials found that tree nuts at a median dose of 56 g/day can improve markers of glycemic control in individuals with type 2 diabetes (including lowering HbA1c and fasting glucose) [45]. Another systematic review and meta-analysis of 40 RCTs at a median dose of 52 g/day, including diverse populations of adults (including healthy, those with type 2 diabetes or with CVD risk factors), found that tree nuts or peanuts improved markers of insulin sensitivity, however, the effect on fasting blood glucose and HbA1c was not significant [46]. Other potential mechanisms include their role in adiposity [47], possibly due to their satiating effect, increased efforts and/or time of mastication and hence incomplete digestion in the intestines, and alpha-linolenic acid content of nuts, especially walnuts, which might increase membrane fluidity of endothelial cells with the enhancement of nitric oxide synthesis and ensuing improvement of endothelial function [48,49]. Overall, there is good evidence that nut consumption consistently lowers atherogenic blood lipids and may improve insulin sensitivity and endothelial function, with less consistent effects on blood pressure, without adversely impacting adiposity. Regarding adiposity, a recent systematic review and meta-analysis highlighted that the median nut intake in the trials included in their analyses, as well as in the health claims noted in Table 2, that a dose of 42.5 g/day could be integrated into a daily dietary pattern without contributing to weight gain [47]. 

## 7. Future Directions

The evidence for nut consumption and total CVD and CHD is more consistent in prospective cohort studies compared to other CVD outcomes, with low to moderate certainty of evidence using the GRADE criteria. The GRADE criteria, however, may not be the best grading system to use when evaluating the certainty of evidence from observational studies, particularly in the field of nutritional epidemiology [50]. Future pooled analyses that assess the certainty of evidence should consider integrating ROBINS-I to assess the risk of bias [51] or consider also applying the NutriGrade system [52], both of which do not provide excessive downgrading of observational evidence. In addition, given the scarcity and sometimes conflicting results among studies, more studies are needed to clarify the role of nut consumption in stroke, particularly stroke subtypes, HF, AF and PAD. Further research on the type of nuts will eventually provide further insight into their role in CVD prevention, including studies of peanut and other nut butters (including natural) and salted vs. unsalted nuts. New analyses on nut intake should also report quantities of nut intake (i.e., grams/day) so that these data can be used in updated meta-analyses. The quantity of nuts is also more translatable for guiding dietary recommendations compared to high vs. low categories. Individual cohort pooled meta-analyses would also be useful to ensure consistency in analyses across cohort studies. Although RCTs of nut consumption and blood lipids support the results from observational cohort studies showing reduced CVD and CHD risk, there is some discrepancy between what doses of nuts have been shown to reduce blood lipids in RCTs and what doses lower CVD and CHD risk in observational cohort studies. For example, in a meta-analysis of RCTs, there was a steeper reduction in total and LDL-C between 50–100 g/d than at lower levels of intake [20], while in the observational cohort studies, maximum risk reductions have been observed around 15–20 g/d (approximately 4–5 servings/week) [21,22,23]. However, the highest nut intakes reported in cohort studies have typically been around one serving/day (28 g/d), and it is unknown whether CVD or CHD risk is reduced further with higher intakes. Considering that relatively few people consume more than one serving of nuts per day in most populations [23,53], pooled analyses may also be needed to explore, with sufficient statistical power, whether higher intakes are associated with further reductions in hard endpoints. Further studies are also needed to clarify if other mechanisms than reductions in lipids (e.g., antioxidant or anti-thrombotic effects) may contribute to the vascular benefits observed at the more modest nut consumption levels reported in the observational studies. Another important consideration related to the discrepancy of nut levels consumed in trials and cohort studies is the dietary assessment tool used, as food frequency questionnaires (FFQ) commonly administered in cohort studies may not be as accurate in quantifying absolute intake compared to trials, where diet records are typically used, and intervention groups are usually provided nuts to guarantee the desired consumption. As diet records are not feasible in large cohort studies, repeated measurements of FFQs will be important to represent long-term dietary habits and reduce measurement error, as well as allow assessment of change in nut consumption in relation to health outcomes. FFQs should also consider including more nut categories (walnuts, almonds, peanuts, seeds, etc.) to provide more detailed information on nuts and nut types. 

Using objective biomarkers of nut consumption alongside dietary intake assessment methods will additionally be important in the future, as they are less prone to measurement error from FFQs or 24-h recalls [54]. For example, in the PREDIMED study, plasma alpha-linolenic acid (a polyunsaturated fatty acid that abounds in walnuts) levels were measured to confirm adherence in the group receiving mixed nuts alongside an FFQ [39]. Novel approaches, such as multi-omics, will likely play a larger role in the future for both assessing adherence to diet and precision nutrition [55]. Metabolomics, in particular, is a promising technique to help identify objective dietary biomarkers by providing a comprehensive representation of overall dietary intake by measuring the metabolites in biological samples (such as blood or urine). In prospective cohort studies, several metabolites, mainly lipid-related, have been found to be markers of nut intake in general [56] or of specific types of nuts, such as walnuts, and these metabolites have likewise been associated with a lower risk of CVD [57]. The metabolites associated with nut consumption may be helpful in identifying potential objective biomarkers of exposure to nuts in large prospective cohort studies, as well as in clarifying underlying mechanisms implicated in disease risk. Importantly though, these metabolomic profiles associated with nut consumption are not highly correlated with self-reported nut consumption and also reflect the metabolic response to consumption and are therefore not completely sensitive or specific markers. Many of these metabolites may also not be able to distinguish between different types of nuts. Thus, dietary intake assessment methods such as FFQs will still be important to determine more specific information on nut consumption. 

Other future directions include undertaking large CV outcome trials of nut-containing dietary patterns, as was previously done with PREDIMED [39] and the current ongoing PREDIMED-Plus trials [58] (both primary CVD prevention trials) and the CORDIOPREV trial (a secondary CVD prevention trial) [59]. One limitation of these trials is that they cannot separate the effect of the Mediterranean diet from that of nuts on health outcomes. Thus, any further trials in this setting could benefit from having an additional intervention group on a Mediterranean diet only (without nuts). Large trials should also consider long-term measurements of renal function, as this area has been given insufficient attention and kidney dysfunction has been causally related to CHD risk [60], therefore highlighting the need for preventative approaches to also preserve renal function. Furthermore, determining metabolomic signatures that can reflect adherence and metabolic response to these nut-containing dietary patterns should be included within these types of trials. This method was previously assessed using the Mediterranean diet in the PREDIMED study, where a metabolic signature that robustly reflected adherence and metabolic response to the diet was determined [61]. The metabolic signature was then used to assess associations with CVD risk and showed stronger inverse associations with CVD risk compared to dietary intake alone in a Spanish and three US cohorts. Mendelian randomization analyses also showed that the genetically inferred metabolic signature was significantly associated with a lower risk of CHD and stroke [61]. These novel approaches hold promise for an objective and complete evaluation of both adherence and metabolic responses to diet, including nuts, and may allow more effective and individualized approaches to dietary interventions in the future; however, further research is also needed in this area. Overall, a combination of efforts, including well-conducted large prospective cohort studies, large RCTs of hard CV endpoints and incorporation of multi-omics approaches and genetics, will help us better understand the role of nuts in the primary and secondary prevention of CVD. 

## 8. Summary and Conclusions

The overall findings and conclusions from this review are that nuts are a beneficial food for CVD risk reduction, with consistent findings for the benefit for total CVD and CHD in prospective cohort studies and likely benefits for stroke and AF, but additional research is needed for HF, AF, PAD and stroke subtypes. Considering all evidence from mechanistic studies, RCTs of intermediate risk factors and CV events, and prospective cohort studies, at least one serving per day of nuts should be considered for CV risk reduction, although further research on the optimal dose is needed. The type of nut may not be important, though more research is needed to confirm this finding. However, a general nut recommendation will provide more variety of options and be important for any availability and affordability concerns for consumers. 

In conclusion, future research is urgently needed as outlined above, particularly for stroke subtypes, PAD and HF, and include individual cohort pooled analyses, large RCTs of nut-containing dietary patterns and using -omics methodologies to better capture adherence and metabolic responses to diet, including the consumption of nuts. 

## Figures and Tables

**Table 1:** Examples of Recommendations for Nuts in CVD Clinical Practice Guidelines.

Guideline Association	Nuts Recommendation
American Heart Association 2021 [10]	“Choose healthy sources of protein, mostly protein from plants (legumes and nuts)”
European Society of Cardiology and European Atherosclerosis Society 2019 [11]	“Food choices to lower low-density lipoprotein cholesterol and improve the overall lipoprotein profile are nuts and seeds”
Canadian Cardiovascular Society 2016 [12]	“We suggest that all individuals be encouraged to moderate energy (caloric) intake to achieve and maintain a healthy body weight (Conditional Recommendation; Moderate-Quality Evidence) and adopt a healthy dietary pattern to lower their CVD risk: Dietary patterns high in nuts (30 g/day) (Conditional Recommendation; Moderate-Quality Evidence)”
Joint British Society Consensus for prevention of Cardiovascular Disease 2014 [13]	“Consider regular consumption of whole grains and nuts”
Heart Foundation Australia 2019 [14]	“Eating patterns for heart health are based on: A variety of healthy protein sources including fish, seafood, lean meat and poultry, legumes, nuts and seeds Healthy fat choices with nuts, seeds, avocadoes, olives and their oils for cooking”

Abbreviations: CVD = cardiovascular disease.

**Table 2:** Examples of Regulated Health Claims for Nuts and CVD Risk Reduction.

FDA	EFSA	FSANZ	
1.5 ounces per day of nuts, as part of a diet low in saturated fat and cholesterol and not resulting in increased intake of saturated fat or calories may reduce the risk of CHD [15]		30 g per day of walnuts may improve endothelium-dependent vasodilation [17]	General level health claim around heart health without causing weight gain allowed for tree nuts, peanuts, ground nuts/butters/pastes [16]

Abbreviations: EFSA = European Food Safety Authority; FDA = Food and Drug Administration; FSANZ = Food Standards Australia New Zealand.

## Data Availability

Not applicable.
